# Transcriptomic analysis of human sensory neurons in painful diabetic neuropathy reveals inflammation and neuronal loss

**DOI:** 10.1038/s41598-022-08100-8

**Published:** 2022-03-18

**Authors:** Bradford E. Hall, Emma Macdonald, Margaret Cassidy, Sijung Yun, Matthew R. Sapio, Pradipta Ray, Megan Doty, Pranavi Nara, Michael D. Burton, Stephanie Shiers, Abhik Ray-Chaudhury, Andrew J. Mannes, Theodore J. Price, Michael J. Iadarola, Ashok B. Kulkarni

**Affiliations:** 1 Functional Genomics Section, National Institute of Dental and Craniofacial Research, National Institutes of Health, 30 Convent Drive, Room 130, Bethesda, MD 20892 USA; 2 Yotta Biomed, LLC, Bethesda, MD 20814 USA; 3 Department of Perioperative Medicine, Clinical Center, National Institutes of Health, Bethesda, MD 20892 USA; 4 Department of Neuroscience and Center for Advanced Pain Studies, University of Texas at Dallas, Richardson, TX 75080 USA; 5 Neuroimmunology and Behavior Group, School of Behavior and Brain Sciences, University of Texas at Dallas, Richardson, TX 75080 USA; 6 Surgical Neurology Branch, Disorders and Stroke, National Institute of Neurological, National Institutes of Health, Bethesda, MD 20892 USA; 7 Present Affiliation: NIH Graduate Partnerships Program, Brown University, Providence, RI 02912 USA

**Keywords:** Neuroscience, Physiology

## Abstract

Pathological sensations caused by peripheral painful neuropathy occurring in Type 2 diabetes mellitus (T2DM) are often described as ‘sharp’ and ‘burning’ and are commonly spontaneous in origin. Proposed etiologies implicate dysfunction of nociceptive sensory neurons in dorsal root ganglia (DRG) induced by generation of reactive oxygen species, microvascular defects, and ongoing axonal degeneration and regeneration. To investigate the molecular mechanisms contributing to diabetic pain, DRGs were acquired postmortem from patients who had been experiencing painful diabetic peripheral neuropathy (DPN) and subjected to transcriptome analyses to identify genes contributing to pathological processes and neuropathic pain. DPN occurs in distal extremities resulting in the characteristic “glove and stocking” pattern. Accordingly, the L4 and L5 DRGs, which contain the perikarya of primary afferent neurons innervating the foot, were analyzed from five DPN patients and compared with seven controls. Transcriptome analyses identified 844 differentially expressed genes. We observed increases in levels of inflammation-associated transcripts from macrophages in DPN patients that may contribute to pain hypersensitivity and, conversely, there were frequent decreases in neuronally-related genes. The elevated inflammatory gene profile and the accompanying downregulation of multiple neuronal genes provide new insights into intraganglionic pathology and mechanisms causing neuropathic pain in DPN patients with T2DM.

## Introduction

The most common form of neuropathic pain arises from Type 2 diabetes mellitus (T2DM)^1^. Approximately 50% of T2DM patients will develop some form of neuropathy, with half of those neuropathic cases considered painful (diabetic peripheral neuropathy—DPN)^2^. Overall, pain is often considered the most bothersome symptom for patients with diabetic neuropathy, reaching ratings of 6 to 10 on a 0 to 10 pain intensity scale^3,4^. DPN is characterized as a length-dependent neuropathy, where distal extremities such as the hands and feet are predominantly affected by pain, thereby producing a characteristic “glove and stocking” pattern^1^. The soma of the primary afferent neurons that innervate the feet are known to reside in the lumbar dorsal root ganglia (DRG). Aberrant peripheral nociceptor firing is implicated in promoting pain hypersensitivity in individuals with DPN, as a local anesthetic nerve block prevents spontaneous pain in these patients^5^. Even though nociceptor hyperactivity is linked to heightened pain sensitivity, drugs currently available that directly target the nervous system often have harmful side effects. Overall, about 72% of DPN patients report that their pain worsens after the initial onset^4^.

Research into understanding DPN is complicated by the fact that diabetes is a multifactorial disorder. Chronic diabetic hyperglycemia can cause oxidative stress by both an enzymatic means through activation of aldose reductase in the polyol (sugar alcohol) pathway and with the non-enzymatic production of advanced glycation end products (AGE) via sugar addition onto proteins^6^. Over time, the accumulating metabolic insult to sensory neurons overrides the capacity for regeneration and repair^1,7,8^. Patients with DPN generally present a heterogeneity of neuropathic symptoms with varying effects on somatic sensation^9^. Additionally, some patients with DPN also develop autonomic neuropathy^10^.

To identify potential gene regulatory processes in humans that can contribute to DPN, we acquired DRGs from 5 DPN patients and 7 non-diabetic controls at the time of organ donation. Gene profiles of lumbar DRGs were generated by RNA sequencing (RNA-seq) analyses along with in situ hybridization and standard histological examinations of the DRGs to investigate potential pathophysiological changes underlying DPN. Innate inflammatory pathways appear to be upregulated in the DPN individuals, possibly in response to cell injury and/or death via alarmins. In contrast, we observed varying degrees of neuronal loss in four out of five of the DPN subjects that lead to coexisting declines in the expression of neuronally enriched genes. Our transcriptomic analysis identified an increase in inflammation within the DRGs of individuals with DPN along with an accompanying decrease in neuronal transcript levels that may reflect phenotypic modification of neuronal gene expression, outright loss of neurons, or a combination of both.

## Results

### Comparative transcriptomic analysis of DPN versus controls

To gain molecular insight into causes of diabetic neuropathic pain in patients, we recovered DRGs from consenting organ donors who were experiencing diabetic painful neuropathy (DPN, Table 1). The sensory neurons of the DRG seem sensitive to the metabolic imbalances occurring with T2DM as compared to other neurons within the peripheral nervous system^1^, yet, only a few studies have used DRGs of patients with diabetes^11,12^. Instead, research into nociception for the past decades has mostly depended on studies in rodents^13^, where studies on diabetic neuropathy primarily involve either animals with nerve injury or animals with experimental diabetes^12^. Although far less pain research has been done on human DRGs, organ-donor networks have now made human DRGs more available, which has allowed for transcriptomic ^14–16^, proteomic^17^, and electrophysiological^18^ studies that compare the characteristics of human DRGs to those of research animals. DRGs from individuals with chronic pain have also become available for research purposes, making our study comparing DRGs from cohort of 5 DPN donors to 7 non-diabetic controls possible (Figure S1). Though limited (further medical details about the DRG donors along with histological scoring are given in the Methods, Table S1, and Table S2), an in-depth analysis of the gene expression changes occurring in the soma of the human sensory neurons is needed to better understand the neuropathological changes occurring with DPN, particularly as rodent models do not adequately replicate human diseases^13,19^.

**Table 1:** Demographics on the DRG donors used in this study. BMI = Body Mass Index, COD = Cause of Death.

Doner	Age	Sex	BMI	Ethnicity	COD
DPN1	58	F	20.1	Caucasian	CVA/ICH/Stroke
DPN2	69	F	28	Caucasian	CVA/ICH/Stroke
DPN3	50	F	43.9	Hispanic/Latino	Anoxia/Cardiovascular
DPN4	59	M	34.2	Hispanic/Italian	Anoxia/Cardiovascular
DPN5	47	M	25	Hispanic/Latino	CVA/ICH/Stroke
Con1	30	F	19.9	Hispanic	CVA/Stroke
Con2	49	F	36.7	Caucasian	MVA/Head Trauma
Con3	39	F	25	African American	CVA/Stroke
Con4	45	M	25.5	Caucasian	Anoxia
Con5	47	M	28.5	Caucasian	Head Trauma
Con6	54	M	23.9	Caucasian	Head Trauma/Blunt Injury
Con7	55	M	21.1	Caucasian	Head Trauma

**Figure 1:**
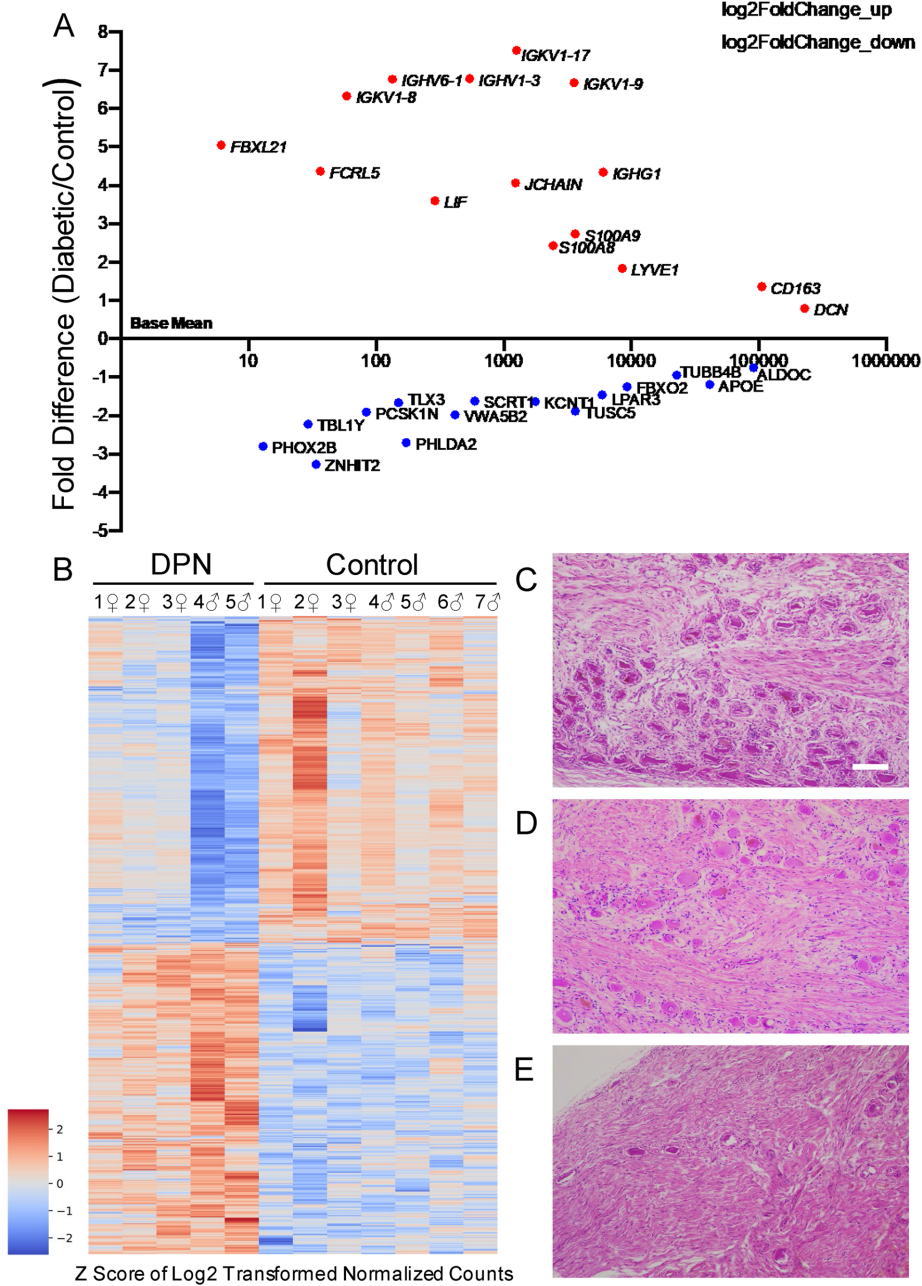
DPN Transcriptome: (**A**) Scatterplot of all DEGs where dysregulated genes with high log2 fold change per base mean of normalized DESeq2 counts are highlighted. Immunoglobulins and other inflammatory related genes are upregulated while some genes involved in neurogenesis like the transcription factors *SCRT1* and *TLX3* were downregulated in the diabetic individuals. (**B**) Heatmap of all 844 DEGs. (**C**-**E**) H and E images of the L4 DRG, scale bar = 500 µm; (**C**) Normal dorsal root ganglion with adequate density of ganglion cells surrounded by satellite cells, (**D**) DPN with a neuropathologist report of moderate loss of neuronal ganglion cells with replacement reactive fibrosis, and (**E**) DPN with a neuropathologist report of severe loss of neuronal ganglion cells with replacement fibrosis (hematoxylin and eosin stains at 40X magnification).

As DPN is a length-dependent neuropathy, affecting the distal extremities such as the fingers and toes, L4 and L5 DRGs were recovered as these ganglia contain the soma of the primary afferent neurons innervating the foot. Bulk RNA-seq was conducted to holistically analyze expression changes in the DRG that can occur with DPN, as the DRG is comprised of not only sensory neurons, but also Schwann cells, satellite glial cells, fibroblasts, and immune cells^14^. Differential gene expression analysis was performed using DESEQ2^20^. With principal component analysis, the DRG donors were initially clustered by sex, indicating that sex strongly affected gene expression in the human DRGs. However, after treating sex as a covariate, we had a clear separation between DPN versus non-diabetic controls as the principal division amongst the donors, ensuring the differences captured by RNA-seq data reflects differences between the DPN and control groups as opposed to another moderating factor, such as sex (Figure S2). Overall, between the two groups, we were able to derive 844 differentially expressed genes (p-value < 0.05), with 411 genes significantly upregulated in donors with DPN versus 433 genes significantly downregulated (Fig. 1, Table S3-5).

In Fig. 1A, we saw that the upregulated genes detected in our transcriptomic analysis include immunoglobulins in conjunction with other inflammatory response genes, while neuronally related genes were essentially downregulated. We analyzed our list of differentially expressed genes (DEGs) with Ingenuity Pathway Analysis (IPA) to determine which biological processes were most affected by diabetic neuropathy, dividing our data set between upregulated and downregulated genes. Analysis of the upregulated genes emphasized the increased inflammatory responses within the DRG of the DPN donors, where the top canonical pathways involve communication between immune cells and diapedesis (Figure S3A). IPA also predicted activation of TNF-α, IL-6, and IL-1β cellular signaling pathways, where these key immunoregulators are known to be associated with diabetes-induced metabolic inflammation^21,22^. We separately examined the downregulated genes and saw that synaptogenesis signaling was predicted to be affected (Figure S3B). Patients with diabetic neuropathy often develop sensory loss over time even while still experiencing pain and our pathway analysis similarly shows a transcriptomic loss of neuronally related gene expression in the DRG of the DPN individuals^10^.

We next histologically examined the L4 DRGs from the donors to see if there were pathological changes that would support our findings from the RNA-seq data (Fig. 1C,E). Of the 5 control DRGs used for histopathological examination, one was mostly normal (Con1), three had mild loss of myelinated axons (Con 3, 6, and 7), while one individual (Con4), with Lennox-Gastaut Syndrome, had mild to moderate neuronal loss. In contrast, a broad span of neuropathology was seen with the DPN individuals (Fig. 1D,E). One female (DPN3) was apparently normal, another female (DPN2) showed mild to moderate neuronal loss, and a third female (DPN1) had moderate neuronal loss with scattered degenerating neurons. Of the two DPN males, DPN4 showed moderate loss of neurons with scattered degenerating neurons while DPN5 had moderate to significant neuronal loss (histological scoring of the donors is presented in Table S2). Greenbaum et al.^11^ also reported a varied extent of pathology in DRGs from diabetic neuropathy patients experiencing pain that ranged from minor degenerative changes to a loss of neurons and aggregates of phagocytic cells in one female patient.

A heatmap of DEGs in Fig. 1B depicts not only the contrast between the DPN patients versus the controls, but also illustrates the range of gene expression changes within the DPN DRGs as well, probably due to differences in disease progression. The heatmap shows that there is evident downregulation in the expression of several genes in the two DPN males that likely stems from advanced neuronal loss. Two of the three female DPN subjects show a loss of neurons as well, but don’t exhibit the same degree of gene expression change as the two males. Nonetheless, the gene expression decreases in these female DPN donors probably still result from neurodegeneration within the DRG. Of note, patients with diabetic neuropathy often show a reduction in distal epidermal nerve fiber density^10^, and this axonal degeneration probably also impacts neuronal gene expression in the sensory neurons. Sex is known to differentially affect both diabetic onset and pain intensity^23^, but, with our limited sample size, we cannot confirm if there is a true sex-linked difference causing the distinct male and female gene expression changes as shown in Fig. 1B or if this pattern is just a consequence of variation in disease progression among the donors. Despite this range in neuropathic symptomology, our transcriptomics is still able to identify common trends amongst the individuals with DPN.

### Transcriptional regulators, ion channels, kinases, and G-protein coupled receptors

**Figure 2:**
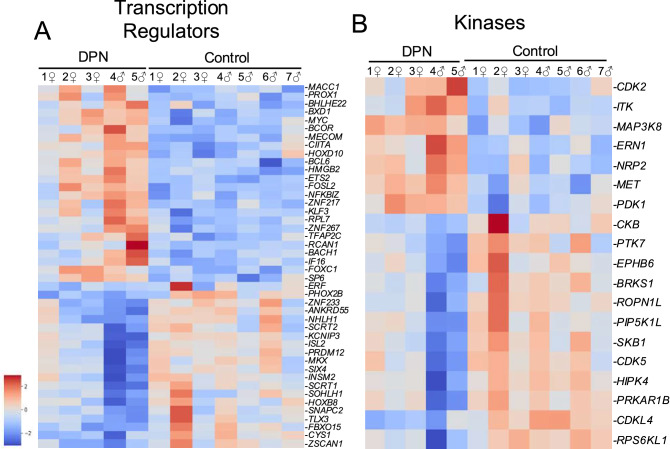
Heatmaps of dysregulated genes belonging to functional protein classes associated with pain signaling. Pictured are heatmaps of (**A**) transcriptional regulators and (**B**) kinases. Inflammation can cause gene expression changes in nociceptors through downstream intracellular signaling cascades that ultimately activate various transcription factors. Inflammatory signaling can additionally stimulate protein kinases that can then modulate pain transducing ion channels to induce peripheral sensitization.

Pain hypersensitivity often occurs downstream of inflammation, where inflammatory mediators are known to activate transcription factors and trigger kinase activity in nociceptors, so we specifically examined the expression changes in these protein classes to search for differences between the DPN individuals and the controls that might impact neuropathic pain (Fig. 2). For transcriptional regulators (Fig. 2A), we saw an upregulation of known T-cell induced transcriptional activators in the DPN individuals including Nuclear Factor Interleukin 3 Regulated Protein (*NFIL3*) and Interferon Gamma Inducible Protein 16 (*IFI16*). We additionally saw that BCL6 Transcription Repressor (*BCL6*) and its corepressor *BCOR* are increased in the DPN donors, which may be indicative of inflammatory cell infiltration. In contrast, we observed downregulation in several transcriptional regulators that are enriched in nociceptors. Transcription factors like *ISL2*, *TLX3*, and *PRDM12*, for example, were all significantly downregulated in the DPN donors^24^. Other downregulated neuronal transcription factors include *NHLH1*, *SCRT1*, *SCRT2*, and *SIX4*.

When examining for changes in kinase expression, inflammation-related genes again were upregulated in the DPN individuals, including *ITK* and *MAP3K8*, both of which are commonly expressed by T-cells (Fig. 2B). Conversely, three creatine kinases were downregulated in the DPN individuals, including *CKB*, *CKMT1A*, and *CKMT1B*, all of which could impact energy utilization in neurons. The expression of *CDK2*, a key cell cycle kinase, is increased in the DPN individuals and could broadly suggest either local immune cell activation, tissue repair, or neuronal apoptosis. Lastly, *NRP2*, a kinase linked with axonal regeneration^25^, was upregulated in the individuals with DPN.

**Figure 3:**
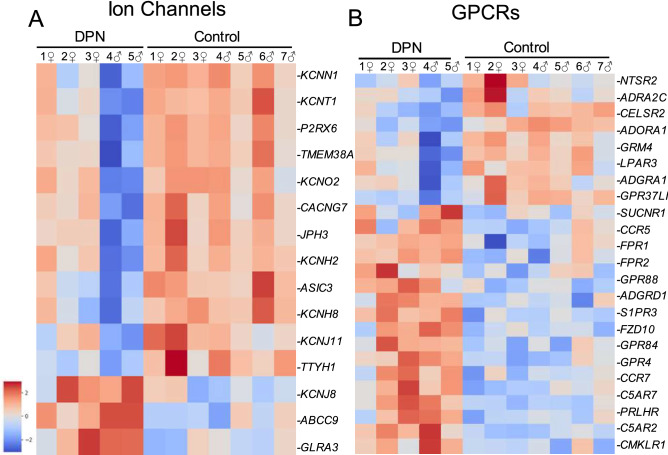
Heatmaps of genes dysregulated in DPN individuals that encode for (**A**) ion channels and (**B**) G protein coupled receptors. Ion channels and GPCR signaling can affect nociceptor firing. Transcription factors can alter the expression of key ion channels and GPCRs that can subsequently promote nociceptor hyperactivity. Kinases can modify the activity of ion channels and GPCRs through post-translational phosphorylation to then regulate the sensitivity to noxious stimuli in nociceptors.

Since nociceptor firing can be modulated by ion channel activity and through GPCR signaling, we next examined gene expression differences within these two protein classes (Fig. 3). With few exceptions, most altered ion channels were downregulated (Fig. 3A). Almost half of the downregulated ion channels in the DPN individuals were neuronally expressed potassium channels, including the potassium voltage-gated channels *KCNH2* and *KCNQ2* (both predominantly expressed in Aβ-fibers), the inwardly rectifying channel *KCNJ11*, the calcium-activated channel *KCNN1*, and the sodium-activated channel *KCNT1*. In addition to these potassium channels, two potassium channel-interacting proteins, *KCNIP2* and *KCNIP3* were downregulated within our dysregulated gene list as well, both of which are calcium binding proteins known to affect neuronal excitability. The downregulation of these potassium channels could potentially lead to either more neuronal hyperexcitability or could simply be indicative of the neuronal loss. Besides the potassium channels, *ASIC3* (acid sensing ion channel 3), a prominent pain transducing ion channel expressed in C-fibers, was downregulated in the DPN donors, while *GLRA3*, a glycine receptor that could contribute to prostaglandin E2 related pain hypersensitivity, is surprisingly upregulated^26^.

Amongst GPCRs, inflammation-related signaling pathways were once more significantly upregulated as neuronal modulatory genes were downregulated in the DPN individuals (Fig. 3B). *C5AR1* and *C5AR2*, receptors for the proinflammatory chemoattractant complement C5a, were both upregulated in the DRGs from the DPN donors. In terms of pain signaling, the DPN donors have increased expression of sphingosine 1-phosphate receptor *S1PR3*, which is known to promote mechanical pain partially via closure of KCNQ2^27^. DPN subjects also showed decreased expression of receptors for adenosine (*ADORA1*), norepinephrine (*ADRA2C*), and dynorphin (*OPRK1*), all of which may possess antinociceptive qualities. Downregulation of these receptors can either cause increased nociceptive hypersensitivity or again be related to neuronal loss. With our bulk sequencing, however, the full implication for some of these example gene expression pattern differences cannot be fully understood without further analysis to identify which cell types have been affected.

### Downregulation of neuronal marker genes

**Figure 4:**
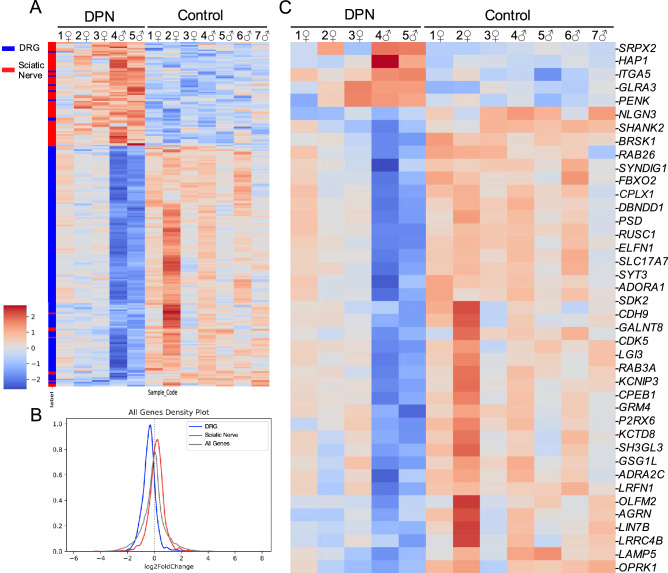
Downregulation of neuronal cell type specific genes: (**A**) Our transcriptomic list of DEGs was compared with a database of rat homologs enriched in the DRG (which contains the soma of sensory neurons) versus those enriched in the sciatic nerve (which predominantly consist of Schwann cells that support descending neuronal axons). (**B**) Neuronally related genes (DRG) were essentially downregulated in the DPN individuals, particularly in the two male DPN donors. Consequently, genes more related to Schwann cell character (sciatic nerve) predominate in the data and are configured as appearing upregulated due to this loss in neuronal gene density. (**C**) Of the neuronally specific genes identified in our list of DEGs, enrichment analysis characterized 41 genes as related to synaptic function.

Along with the neuronal loss seen histologically, our transcriptome analysis also shows a corresponding decrease of additional neuronally-related genes in the DPN donors (Fig. 4). Of particular note is the significant downregulation of the NeuN gene (*RBFOX3*), a well-established neuronal marker. Synuclein Gamma (*SNCG*), a gene highly expressed in the peripheral nervous system, shows decreased expression in the DPN donors as well. *LPAR3*, which is enriched in a subpopulation of *TRPV1* + neurons, and *PVALB*, which labels DRG myelinated proprioceptors, are both additionally downregulated in the DPN individuals and supports the idea that diabetic neuropathy ultimately impacts multiple sensory neuron types^28^. Additionally, the DPN individuals show downregulation of *NAT8L*, a key enzyme known to catalyze the formation of the metabolite N-acetylaspartate (NAA), which is important for neuronal health.

With this transcriptomic loss in neuronal genes, we decided to compare our RNA-seq data with a dataset of genes enriched in the rat DRG and sciatic nerve^14^ (Fig. 4A,B). While the DRG contains both neurons and Schwann cells, the DRG is enriched in neurons while the sciatic nerve is predominately comprised of Schwann cells, which allows for identification of genes differentially enriched in these two cell types. In general, most neuronally enriched genes are downregulated compared to Schwann cell genes. Dysregulated neuronal genes were then characterized according to cellular function. Of the genes categorized as a structural component of a neuron, about 66% were associated with the synapse while 39% are considered as part of the neuronal cell body (Figure S4A). In particular, genes linked to synaptic vesicle exocytosis are downregulated in the DPN individuals including *CPLX1*, *SYT3*, and *RAB3A* (Fig. 4C). Key neuronal kinases that affect neurotransmitter release including *BRSK1* and *CDK5* were downregulated also (Figure S4B). As seen in Fig. 4C, the DPN individuals additionally have decreased expression of *CPEB1*, a regulatory protein that controls the translation of mRNA that is localized to the synapse. Surprisingly, some notable neuronal genes are upregulated in the differential gene expression analysis despite the overall neuronal loss seen in the DPN individuals. Ephexin1 (*NGEF*), for example, is upregulated, possibly in response to neuronal injury^29^.

**Figure 5:**
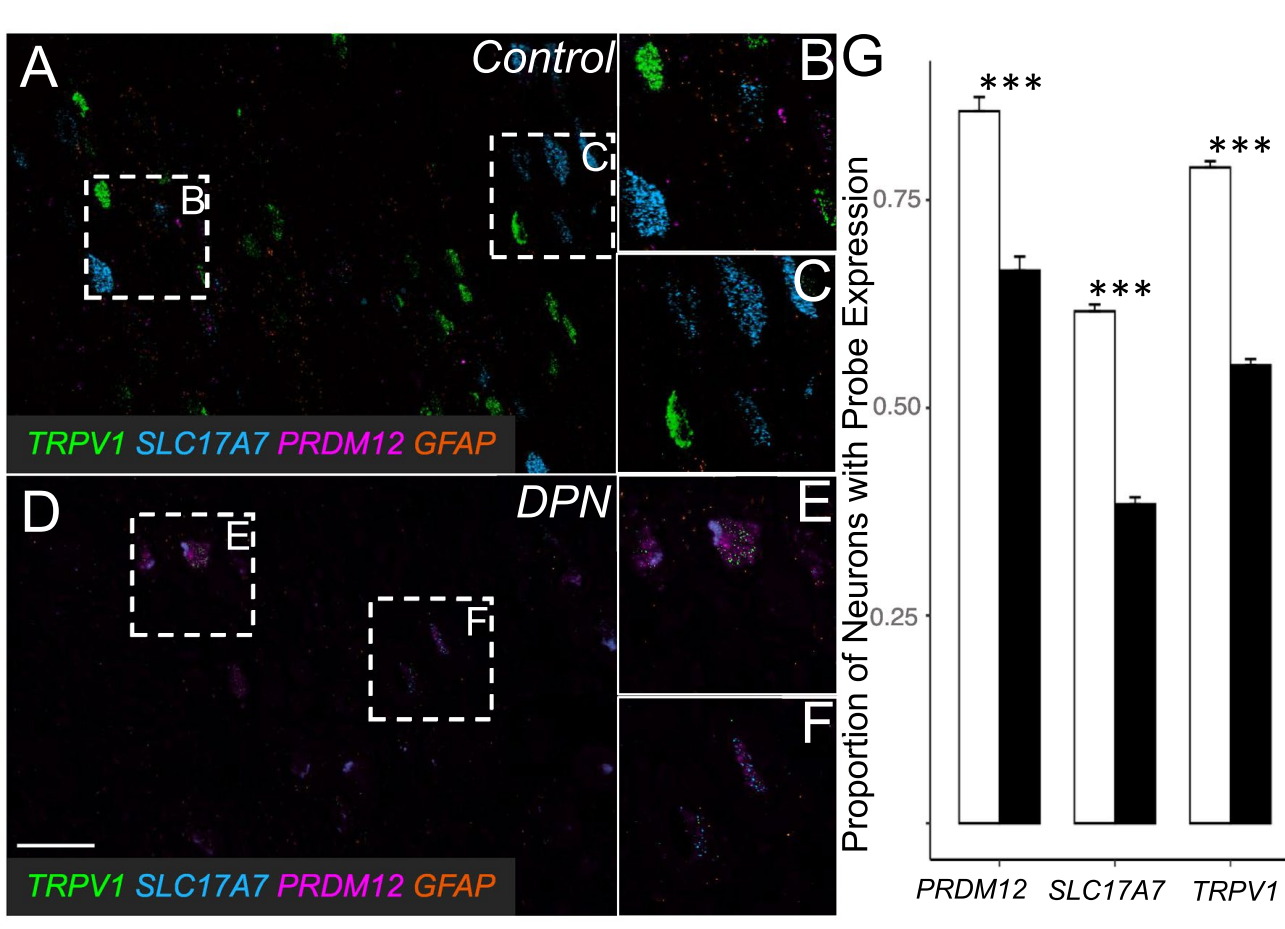
Representative images and comparison of RNAscope data: RNAscope data confirms a loss of neuronally related genes as noted in RNA-seq results by demonstrating a decrease in the proportion of neurons expressing each neuronal probe. (**A**) DRG of non-diabetic individuals displayed (**B**) small diameter *TRPV1* positive neurons and (**C**) larger diameter *SLC17A7* positive neurons. (**D**) DRG of DPN individuals had decreased proportions of neurons expressing all three probes, while still containing expected neuronal subtypes such as (**E**) small *TRPV1* positive and (**F**) larger *SLC17A7* positive neurons. Scale bar = 100 µm. (**G**) Differences in proportion of neurons expressing each RNAscope between control and DPN tissue were significant at the ***p < 0.0001 level, according to the Cochran-Mantel–Haenszel Test (*TRPV1* and *SLC17A7*), and Fisher’s Exact Test (*PRDM12*). Each probe was significantly downregulated, evidence of a loss of neuronally related gene expression in DPN DRG. Data is shown graphically as mean and standard error. The downregulated expression of these three neuronal markers coincides with the neuronal loss seen histologically. Although dedifferentiation could cause a loss of *TRPV1* + and *SLC17A7* + neurons, most neurons were still labelled with either of these two markers. White and black bars represent the control and DPN neurons expressing RNAscope, respectively.

Lastly, to investigate the neuronal loss further, in situ hybridization was performed on sections of L4 DRGs in order to visualize the expression of three neuronally associated genes: *TRPV1*, a known pain transducer highly expressed in nociceptive C-fibers^30^; *SLC17A7*, a glutamate transporter that labels large diameter sensory neurons; and *PRDM12*, a key transcriptional regulator of nociceptors during development (*SLC17A7* and *PRDM12* were both significantly downregulated in our transcriptome analysis). Consistent with the histological report, *TRPV1*, *SLC17A7*, and *PRDM12* were expressed in the DPN individuals in a lower proportion of DRG neurons than in control DRG neurons (Fig. 5). Expression of all three genes was significantly decreased at the p < 0.0001 level. Essentially, the loss of normal neuronal markers demonstrated in transcriptomic results was validated in our ISH fluorescence labeling. Despite the possibility of nociceptive phenotype loss, there are residual primary afferent neurons that could still trigger spontaneous pain^5^.

### Increased inflammation in the DRG due to DPN

**Figure 6:**
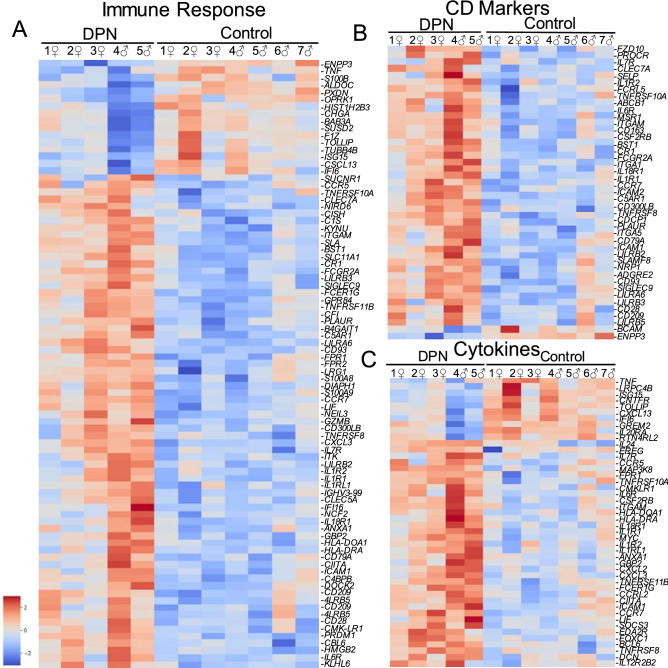
Dysregulated immune response in the DPN individuals: (**A**) Gene enrichment analysis identified 89 genes associated with immune responses in our list 844 DEGs. As shown in the heatmap, most genes were upregulated in the DRG of the DPN subjects while only a few inflammatory genes were downregulated. (**B**) 47 genes were further characterized as involved specifically in cytokine-mediated signaling. (**C**) 44 genes were considered CD markers (HGNC group ID 471).

Our transcriptome analysis shows a downregulation in neuronal marker genes while, conversely, displaying an increased immune cell signature. We see indications of macrophage recruitment to the DRG in DPN patients, which is similarly seen in rodent models of neuropathic pain^31^. We also see the induction of an antibody immune response in the DPN individuals. (Table S6), with immunoglobulins accounting for ~ 17% of all significantly upregulated genes and 39 of the top 100 most significantly altered genes, all of which may reflect an aberrant B cell polyclonal activation that has been reported to be circulating in the blood of patients with diabetes^32^. Transcriptome analysis, however, cannot determine if the immunoglobulin gene products are targeted towards neurons or supporting glia. We also detected upregulation in numerous genes related to immune responses (Fig. 6A). Upregulated inflammatory genes are seen across all DPN individuals, which coincides with the increased metabolic inflammation that develops in patients with diabetes^21,22^. We see, for example, that S100A8/A9 are both increased in the DPN donors, where elevated circulating levels of these proteins can act as a biomarker for diabetes-induced inflammation^33^. S100A8/A9 are mainly produced by neutrophils^34^ and macrophages but may also be expressed by Schwann cells post-injury^35^. Further analysis of the dysregulated inflammatory responses revealed that aspects of innate, humoral, and T-cell immune activity are all upregulated in the DPN individuals (Figure S5).

We next searched for differentially expressed genes that encode both for cluster of differentiation (CD) cell surface markers and cytokines to further characterize the intra-DRG inflammatory process. In Fig. 6B, *TNFRSF8* (CD30), a marker of activated T and B cells was upregulated in the DPN individuals along with *CD93*, which is required for maintenance of antibody secretion^36^. The macrophage marker *PLAUR*^37^ is upregulated in the DPN subjects together with an accompanying increase in *ICAM1* (CD54). Upregulated ICAM1 expression may indicate the recruitment of neutrophils into the DRG, which correlates with the presence of S100A8/9^34^. Accompanying the pro-inflammatory response within the DRG of the DPN subjects, M2 macrophage scavenger receptors including *MSR1* (CD204) and *CD163* were both upregulated in the DPN individuals, where M2 macrophages are known to be involved in the phagocytic clearance of cell debris and the resolution of inflammation, perhaps pointing to a mechanism as to how neuronal debris may be cleared.

In terms of cytokine signaling (Fig. 6C), several members of the interleukin 1 receptor family were upregulated including *IL1R1*, *IL18R*, and *IL1RL1* (IL33R). Also, of interest is the increased expression of *IL6R* in conjunction with elevated *IL1R1* as high circulating levels of both IL1β together with IL-6 are predictive of developing T2DM^22^. In contrast, the DPN donors show increased expression of *IL10*, an anti-inflammatory cytokine that is considered protective against neuroimmune diseases^38^. In terms of chemokine signaling, inflammation in the DRG of the DPN donors appeared to be accompanied by increased expression of the leukocyte chemoattractant ligands *CXCL2* and *CXCL3*, otherwise known as macrophage inflammatory protein 2-alpha and beta. Lastly, the DPN subjects exhibit upregulation of the chemokine receptor *CCR5*, whose increased expression could indicate a shift towards a M1 proinflammatory macrophage status^39^.

**Figure 7:**
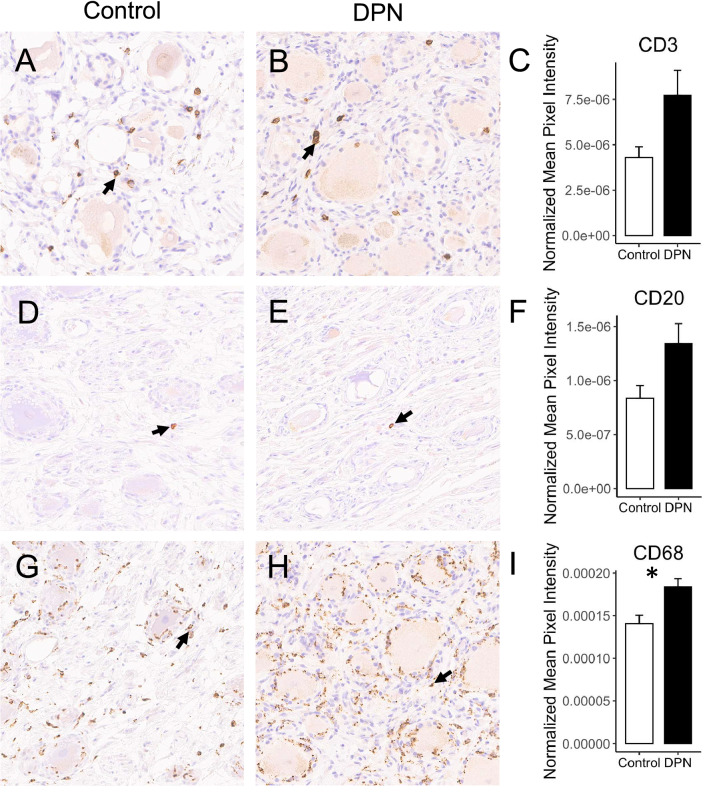
Three CD markers were chosen to characterize the immune related responses observed in the transcriptome: (**A**) and **B**) CD3, a T-cell marker, (**D** and **E**) CD20, a B-cell marker, G and H) and CD68, a macrophage marker. Scale bar = 100 µm. Stained images were scanned and processed using FIJI to quantify average pixel density normalized over the number of hematoxylin-stained nuclei for each image (83). Paired t-tests were performed to compare average expression of CD3, CD20, and CD68 between DPN (**B**, **E**, and **H**) and control DRG (A, D, and G). Average differences in expression were higher for all three markers in the DRG of the DPN individuals, but only CD68 was significant (**C**, **F**, and **I**) (*p < 0.05 level, one-tailed paired t-test), suggesting the activation primarily of innate macrophage driven immune responses.

The presence of inflammatory response genes in our DRG transcriptomic dataset suggests both an activation of resident immune cells along with leukocyte infiltration into the DRG of the DPN donors. To better understand the nature of the increased immune response in DPN DRG (i.e., cell-mediated, humoral, or innate), immunohistochemistry was performed for CD3, a T-cell marker, CD20, a B-cell marker, and CD68, a macrophage marker. The staining intensity for CD3 (Fig. 7A–C) and CD20 (Fig. 7D–F) were both higher in the DPN subjects versus the controls but were not statistically significantly so, even despite the upregulation of B and T-cell markers in our analysis. However, increased staining for CD68 (Fig. 7G–I) was statistically significant within the DRG of those with DPN, demonstrating that the upregulated inflammatory markers captured in the transcriptomics are likely linked to innate immune responses, potentially stemming from diabetes related neuronal pathology. Our immunohistochemistry staining suggests that macrophage-driven inflammation may have a more predominant impact on the DRG of the DPN donors.

## Discussion

T2DM causes axonal loss and pain in about a quarter of patients^1,2^. The metabolic disorder resulting from T2DM can cause hyperexcitability in sensory C-fiber neurons that, in turn, may promote neurodegeneration^2^. Neuronal injury can also affect neighboring “spared” nerve fibers^31^. In general, about 39% of patients with DPN do not receive any treatment for pain^40^, while, of those who are treated, only one-third achieve at least a 50% therapeutic reduction in pain symptoms^41^. Like other types of neuropathic pain, treatment with traditional chronic pain medications such as nonsteroidal anti-inflammatory drugs or opiates is often ineffective in alleviating DPN, and most clinical drug trials have failed to produce any significant therapeutic outcomes^1,42^.

To better understand the underlying pathology associated with painful neuropathy, we performed transcriptomic analysis using DRGs recovered from individuals who had DPN. Through organ donor networks, it has only recently been possible to obtain a small cohort of human DRGs (n = 5) from patients experiencing DPN, which, importantly, are collected within less than 3 h post cross-clamp of the aorta to greatly diminish any post-mortem effects (see further details about the DRG donors are available in the methods and in Supplemental Table S1). For the control group, we asked for control donors with no history of chronic pain, which typically affects an aging population. Although not significant by the Mann–Whitney U test (p = 0.053, Figure S1), our control group tended to be about 10 years younger than the DPN donors. The DRG is a heterogenous tissue, so bulk RNA-seq was conducted to identify possible contributing factors such as microangiopathy, Schwann cell dysfunction, or aberrant macrophage activation. Only a few pain studies have used human DRGs that contain the neuronal soma^14,15^. Our study uniquely uses RNA-seq analysis to compare DRG gene expression differences in non-diabetic controls versus a cohort of subjects that all share the same chronic pain condition. We were also able to make clear connections between the gene expression changes uncovered through RNA-seq with the histopathology seen in the DRG, particularly in terms of neuronal loss and activation of macrophage populations.

Overall, inflammation can be an important factor following neuronal injury, as macrophages are needed to clear cellular debris. Sustained inflammation, however, can be detrimental to neuronal viability^43^. Our transcriptome analysis suggests that aberrant inflammatory processes occur in the DRGs of the DPN donors (Fig. 6A). Low-grade inflammation (“metaflammation”) is generally known to be linked to diabetes^21,44^. For example, monocytes isolated from peripheral blood of T2DM patients have been shown to exhibit higher levels of proinflammatory markers including IL-6, IL-1α, TNF-α, and ICAM1^21^. Although *TNF* (TNF-α) was downregulated, the expression levels of *ICAM1* along with the interleukin receptors *IL1R1* and *IL6R* were all elevated in the DPN donors in our study (Fig. 6A). IL-1β and IL-6 are key regulators of inflammatory reactions and together are predictive for the risk of developing T2DM^22^. IL-1β and IL-6 are both known to stimulate nociceptor firing and are both reported to be upregulated in sural nerve biopsies of patients with progressive diabetic neuropathy^45–47^. Chronic hyperglycemia arising from T2DM can cause the activation of macrophages that then often proceed to infiltrate into adipose tissues. We likewise see increased staining for CD68 in the DRG of the DPN donors (Figs. 7G,H) as well as the upregulated expression of macrophage markers including *PLAUR*, *MSR1*, and *CD163* in our transcriptomic data set (Fig. 6B). The inflammation seen in the DPN donors suggests a reaction to signaling by alarmins that are released in response to cell death^48^. The signature of a macrophage driven immune response was similarly detected in a transcriptomic analysis of the db/db mouse model of type 2 diabetes^49^. Macrophages are known as key regulators of neuropathic pain and depletion of macrophages in rodent models can attenuate hyperalgesia^50^. Besides expressing pro-inflammatory cytokines, macrophages can also contribute to neuropathic pain by recruiting neutrophils to the site of injured neurons, particularly through the release of *CXCL2*, a chemokine that is upregulated in our data set as well^51^. The resident macrophages within the DRG can also play a key role in promoting neuropathic pain along with those in the periphery^52^. Nonetheless, pro-healing M2 macrophages are probably present in the DRG of the DPN donors, too, as suggested by upregulation of the macrophage scavenger receptors *MSR1* and *CD163* and the increased expression of *IL10*.

DRG neurons and their axonal projections lie outside the blood brain barrier and are less protected than neurons within the CNS, but these primary afferent neurons have, in general, a known capacity for axonal regeneration following nerve damage^43^. In our transcriptomic results, some upregulated neuronal genes in the DPN individuals indicate possible attempts at neuronal regeneration including *NRP1* and *NRP2*^25^. However, axonal degeneration probably overrides regeneration, which may account for the predominant transcriptomic loss of neuronal genes within the DRGs of those exhibiting DPN^1,7,8^. In nerve transection mouse models of neuropathic pain, there is a downregulation within the sensory ganglionic neurons of neuron-specific genes, similar to our findings, yet there is also the induction of injury-induced genes like *Atf3*, which was not observed in our transcriptomic analysis of the DRG of DPN individuals, possibly because of the different nature of the neuropathy or the clinical versus experimental time-course^53^. In addition, while the decrease of neuron-specific genes seen in our transcriptomic analysis could partly be explained by the reduced expression of key transcriptional regulators in the DPN patients^24^, the downregulation in neuronal markers may reflect the histopathological findings of neuronal loss in some of the DPN subjects (Figs. 4 and 5). Although neuronal loss could explain the trends observed in RNA-seq and in-situ hybridization data, further investigation is needed to confirm precise underlying mechanisms.

Patients with DPN report experiencing spontaneous pain that is described as sharp and burning and our transcriptomic analysis identified several genes reported to be linked to nociceptive signaling (Tables S7 and S8)^5^. Some aspects of this heightened pain sensitivity may be immune related, as we detected upregulation of multiple inflammatory markers. Interestingly, several potassium channels are downregulated that are known to hyperpolarize a neuron’s membrane potential to inhibit neuronal firing. A decrease in these channels in the DPN individuals may contribute to spontaneous pain sensations (Fig. 3A). Downregulated expression of *KCNQ2* (K_v_7.2), *KCNN1* (SK1), and *KCNT1* (SLACK) in particular are associated with increased sensitivity to pain^54^. The decreased expression of these potassium channels may either promote nociceptor hypersensitivity or be indicative of neuronal loss. A few neuronally related genes are upregulated though, including, for example, *S1PR3* and *GLRA3*, both of which are involved in promoting pain hypersensitivity. Attempts at axonal regeneration may also play a role in causing painful diabetic neuropathy versus non-painful neuropathy^7^. Axonal outgrowth may require the reactivation of some genes associated with neuronal development, so we provide a list of such upregulated genes in Table S7. Surprisingly in our transcriptomic analysis, many of the voltage gated channels, ion channels, and neuropeptides that are involved in pain hypersensitivity were not upregulated in association with DPN even though increased expression of these proteins has been hypothesized to contribute to peripheral sensitization, particularly in rodent models of neuropathic pain^8^.

Pain is commonly experienced in the early stages of disease progression, even at a prediabetic stage^55^. In our study, the DRGs from DPN subjects were collected late in the disease progression, often after years of diabetic symptoms. Thus, our dataset does not examine the initial etiological factors in diabetes that cause pain, but instead examines the long term gene expression differences at the time of death. Our data basically focuses on the gene expression changes within the peripheral neurons of the DRG, but central sensitization also plays an important role in causing chronic pain in the DPN patients^56^. For example, nerve injury or inflammation can cause 2nd order nociceptive interneurons in the dorsal horn to undergo dendritic restructuring that leads to wider receptive fields^57^. Loss of neuronal inputs to the spinal dorsal horn may also shift inhibitory/excitatory circuit balance leading to enhanced pain signaling through remaining nociceptive inputs to the dorsal horn.

With our transcriptomic study, we see an increase in proinflammatory responses with a concurrent loss in neuronally related genes within the DRG of the DPN individuals. Patients with diabetic neuropathy ultimately lose feeling in their legs and feet as subjects who show higher sensory loss often paradoxically report more severe neuropathic pain^8–10^. Our transcriptomic data concur with these reports of sensory loss as there is a decrease in neuronally related genes. Signs of potential neurotoxic processes can be seen in the transcriptomic analyses, for example, with upregulation of the ER stress sensor *ERN1* (Ire1-α) along with *TXNIP*^58^, a regulator of redox signaling. This is also consistent with previous studies showing activation of the ER stress pathway in peripheral nerves in rodent diabetic models and stimulation of ER stress in nociceptors by the diabetic metabolite methylglyoxal^59,60^. The neuronal loss in the DPN subjects could alternatively result from Golgi degeneration, as reported in a rat model of diabetes^61^. In conclusion, our unique approach to transcriptomic profiling of DRGs obtained from diabetic neuropathy organ donors provides new insights into the pathology and mechanisms of DPN. These foundational studies raise new questions that can be explored in future experimental studies.

## Methods

### Human dorsal root ganglia preparation

DRGs used in this study were acquired from the cadaveric donors with consent of the next of kin, as human DRGs are almost exclusively collected post-mortem (Anabios, San Diego, CA) (Table 1). Both L4 and L5 DRGs were chosen as these ganglia contain the soma of the sensory neurons innervating the foot. DRGs were either snap frozen or stored in RNAlater (Ambion, Austin, TX). The DPN donors include 2 males and 3 females (Age: 56.6 ± 3.9 SEM; BMI: 30.2 ± 4.1 SEM), with most reporting diabetic neuropathy for 10 or more years (1 with an incomplete medical history). For our transcriptome study, we both needed a suitable number of human DRGs from patients with DPN to provide significance while also wanting to avoid post-mortem effects, and these criteria have only recently been achievable through organ donor networks. DRGs were collected quickly under cold ischemic conditions, generally 3 h post cross-clamp of the aorta. As organ donors, though, only a limited patient history of diabetes and the severity or progression of associated neuropathy was available. Information about their medical history is provided through an extensive interview with a family member by a trained interviewer (Table S1). Amongst the donors, 3 out of 5 donors were known to have taken medication for pain relief (2 were prescribed analgesic opioids while the other was reported to take enteric aspirin). Despite the limitations in a detailed medical history, organ donor networks are, as of now, essentially the best option for obtaining human DRGs from patients with DPN, whereas a multiregional clinical study that includes pain testing accompanied with questionnaires would be arduous to establish and take a considerable number of years to implement (an example is the longitudinal Religious Orders Alzheimer’s Disease study which has been ongoing for almost 30 years^62^). Unlike previous DPN studies that employ sural nerve biopsies^47^ that are more accessible, our analysis focuses on the DRG that contain the primary afferent neurons as a means to best identify cellular changes within the neuronal cell body itself that may be associated with the axonopathy and demyelination that is often seen in the periphery. The non-diabetic controls used in this study were donors with no known history of chronic pain such as osteoarthritis, back pain, knee pain, etc. The controls, 4 males and 3 females (Age: 45.6 ± 3.3 SEM; BMI: 25.8 ± 2.1 SEM), had cause of death listed as either stroke (CVA) or head trauma. Although statistical analysis showed no significant age difference between the two groups there was some variance between the groups (a trend towards younger age in controls, *p* = 0.053; Figure S1). RNA was extracted from one of the L5 DRGs, while a L4 DRG was used for histology, specifically to look for signs of neuropathy, such as a decrease in neuronal markers^10,61^, and to identify any possible factors that promote neuronal hyperexcitability and pain.

### RNA purification and sequencing

Bulk RNA sequencing was performed to examine differential gene expression in multiple DRG cell types that could contribute to DPN. Additionally, bulk sequencing offers reliable statistical evaluation of the gene expression changes in the DRG that averts sparse input and provides less noise. The L5 DRG was homogenized using a Bio-Gen PRO200 (PRO Scientific, Oxford, CT) rotor–stator homogenizer with Multi-Gen 7XL probes. These were 7 mm sawtooth probes that were detachable so that a clean probe could be used for each sample to avoid cross-contamination. RNA was then purified with a RNeasy Midi Kit (Qiagen, Valencia, CA) using on-column DNase-I digestion according to the manufacturer’s protocol. RNA quality was scored using a Bioanalyzer (Agilent, Santa Clara, CA) and RNA integrity scores (RINs) between 7–9 were obtained (Table S10), indicating some moderate degradation. Libraries were prepared using an Illumina TruSeq mRNA Sample Prep Kit (polyA + method) (San Diego, CA) with 1 µg of total RNA and then sequenced with an Illumina NovaSeq-6000 according to the manufacturer’s protocols at the NIH Intramural Sequencing Center (Rockville, MD). Although the DRGs were collected over a roughly two-year period, library prep and sequencing were performed at the same time to avoid batch variation. Amplification was performed using 10 cycles (to minimize the risk of over-amplification), and unique dual-indexed barcode adapters were applied to each library. Libraries were pooled in an equimolar ratio and sequenced on an S4 flow cell on a NovaSeq 6000 using version 1 chemistry to achieve a minimum of 74 million 150 base read pairs. The data were processed using RTA version 3.3.4.

### RNA-seq analysis

For the human DRG RNA-seq analysis, paired-end sequencing was performed with a read length of the 150 bp. Read quality was checked using FASTQC version 0.11.6. Trimming was performed using BBTools version 38.42 to trim 20 bp off from 5’-end, and 30 bp off from 3’-end. The alignments were performed using STAR version 2.7.2a to the hg38 reference human genome and Gencode release 27 for transcriptome annotation. Read counts per gene per sample were quantified using HTSeq version 0.9.1. Table S10 shows alignment statistics and the total number of reads mapped to genes per sample. A list of DEGs was generated using DESEQ2 version 1.24.0^20^. Sex of the donor was treated as a covariate in the design when building the generalized linear model for DESEQ2 analysis, hence, removing unwanted variation. With adjusted p-value cutoff of 0.05 by Benjamin Hochberg’s False Discovery Rate (FDR), differential expression returned 411 genes up-regulated genes and 433 genes down-regulated genes in the diabetic neuropathy condition compared to the non-diabetic controls (Tables S3-5). Principal component analyses were performed before and after the covariate controlling of sex of the donor, which show improved separation of DPN vs. Control along PC1 axis (Figure S2).

Our study is a detailed examination of transcriptomic signatures of DRG in individuals affected by DPN, however, our results should be interpreted with some limitations. While we demonstrate significance using a small cohort, that we believe is representative, the study cannot capture the true heterogeneity of the complex subtypes of DPN. These initial findings may be useful for guiding larger studies with more patients, but these efforts could take years to come to fruition. While sex differences in pain have been previously established, it was not feasible to divide the two groups by sex in our cohort. Rather, we have controlled for variation due to sex within the RNA-seq analysis pipeline and have observed that two male patients showed stronger responses in terms of transcriptional signatures (Figure S2). However, a larger sample would be needed to understand the various factors (sex, age, weight, disease progression) that fully characterize variation in DPN subjects. In addition, age was found to be only marginally non-significantly different between the two groups (Fig. S1, p = 0.053, Mann–Whitney Test). When using adult DRGs, age is not expected to cause large gene expression changes, however, it is a variable that could interact with disease progression and should be examined in future studies. Information about donor pain associated with DPN and medication use was reported by next of kin and showed some variation from person to person. For example, 3/5 DPN donors were reported to have been taking medication to treat T2DM (i.e., insulin, antihyperglycemics, or insulin response enhancers). The drug history of each patient may also be another factor to examine along with disease progression in a larger cohort. Finally, another interesting component to examine in future studies would be the correlation of molecular changes such as those described here, and behavioral pain outcomes such as pain ratings, pain modalities and a score for pain interference, all of which would help understand the interactions between molecular changes and clinical impact.

### Pathway and data analysis

The upregulated and downregulated dysregulated genes were separately examined with Ingenuity Pathway Analysis (Qiagen, Valencia, CA), due to the robust upregulated inflammatory signal detected in the DPN donors. The STRING Database (Version 11.0) (https://string-db.org/) and ToppGene Suite (https://toppgene.cchmc.org) were also used to identify interactions between significantly differentially expressed genes. Of the 593 recognized genes in STRING, enriched Gene Ontology (AMIGO)^63^ terms included immune response GO:0,006,955 (89 genes with a false discovery rate of 8.17e-06) and cytokine-mediated signaling pathway GO:0,019,221 (47 genes; false discovery rate of 5.09e-05). Heatmaps (generated with Seaborn in Python, version 3.8.3) display the Z Score of Log2 Transformed Normalized DESEQ2 Gene counts. In order to further evaluate DRG compositional changes resulting from DPN, the DEGs from the human donors were compared with bulk RNA sequencing of rat DRG and sciatic nerve, which serve as surrogates for peripheral neurons and Schwann cells respectively^14^. A heatmap was created to display the counts of the DPN human sample sequencing counts for the overlapping gene list, while also labeling the genes enriched in the neuronal or Schwann cell group. The Z Score by gene of the Log2 corrected counts are displayed. The density plot was created with the log2 corrected fold change for all genes that overlap from the enriched for neuronal or Schwann cell gene sets (not just the differentially expressed). This shows the trend towards up or downregulation amongst the entire gene enrichment list. In order to calculate the likelihood of the bias of the enriched datasets towards up or downregulation in the human DRG dataset, a hypergeometric test was performed for each enrichment gene list. A scatterplot of the DEGs was additionally generated using GraphPad Prism (Version 8.0.2) to compare log2 fold change to base mean of normalized DESeq2 counts.

### Histology and immunohistochemistry

Frozen L4 DRGs were temporarily held at -20^O^C for cutting purposes, sliced in half, and immediately placed in 10% buffered formalin (overnight). DRGs from 5 control and 5 DPN donors were then sent for paraffin embedding and sectioning (Histoserv, Germantown, MD), where 6 μm sections were stained with hematoxylin for histological analysis. A pathologist scored blinded slides from the control and DPN donors on a scale of 0 to 3, with 0 being no ganglionic cell loss/within normal limits and 3 being severe cell loss. Table S2 demonstrates these differences in pathological scoring. Immunohistochemistry was also performed by Histoserv (Germantown, MD) for CD3 (1:100 dilution—AB17143 CD3 antibody [F7.2.38], Abcam, Cambridge, MA), CD20 (1:100 dilution—AB9475 CD20 antibody [L26]), and CD68 (1:500 dilution—AB783, CD68 antibody [PG-M1]). Slides were deparaffinized and then subject to either citrate-based (CD20), or tris/EDTA-based (CD3 and CD68) antigen retrieval. Following incubation with primary antibody, immunodetection was performed with a polymer-based reagent. Stained slides were scanned on an Aperio AT2 imaging system (Leica Biosystems, Buffalo Grove, IL) and images were evaluated using FIJI to quantify average pixel density normalized over the number of hematoxylin-stained nuclei identified by the program for each image^64^. Paired t-tests were used to determine differences in CD marker expression between DPN and control DRG.

### RNAscope analysis

Formalin fixed paraffin embedded (FFPE) sections from the L4 DRG were used for in situ hybridization (5 controls and 5 DPN donors). Three neuronal genes (*TRPV1*, *SLC17A7*, and *PRDM12*) were labelled using RNAscope technology^65^. Using custom software, paired double-Z oligonucleotide probes were designed against target RNAs, as follows: Hs- TRPV1-C3, cat no. 415388-C3, NM_080706.3, 20 pairs, 1922–2905; Hs-SLC17A7-C2, cat no. 415618-C2, NM_020309.3, 20 pairs, nt 1311—2570; and Hs-PRDM12, cat no. 559968, NM_008509.2, 20 pairs, nt 117–1789. *TRPV1* and *SLC17A7* probes were stained for in five and four different images per donor (respectively), while *PRDM12* was stained for in just one image for each donor. The RNAscope LS Multiplex Fluorescent Reagent Kit (Advanced Cell Diagnostics, Newark CA) was used on FFPE sections according to manufacturer’s instructions, with target retrieval using ER2 buffer at 95 °C for 15 min, and protease III at 40 °C for 15 min as pre-treatment. Each subject was quality controlled for RNA integrity using an RNAscope Human 4-plex Positive Control Probe (Advanced Cell Diagnostics, Newark CA). Fluorescent images were acquired with a 3D Histech Scanner at 40 × magnification. For each scanned image, three neuronally dense areas were selected for analysis. Three reviewers naïve to tissue sample condition evaluated all neurons in these areas for expression of *TRPV1, SLC17A7*, and *PRDM12*. Cochran-Mantel–Haenszel Tests, which can be utilized for repeated tests of independence, were performed for two probes (*TRPV1* and *SLC71A7*), and Fisher’s Exact Test was performed for one probe (*PRDM12*) in determining differences in the proportions of DRG neurons expressing each probe. The Cochran-Mantel Haenszel test was chosen to test for significance in *TRPV1* and *SLC17A7* expression as to allow the data from five and four (respectively) images per donor to be amassed. Additionally, Fisher’s Exact Test was chosen for *PRDM12* as only one image per donor was stained for this probe.

## Supplementary Information

Supplementary Information 1.

Supplementary Information 2.

Supplementary Information 3.

Supplementary Information 4.

Supplementary Information 5.

Supplementary Information 6.

Supplementary Information 7.

Supplementary Information 8.

Supplementary Information 9.

Supplementary Information 10.

Supplementary Information 11.

## Data Availability

All datasets are available through the dbGaP with accession code- phs002548.v1.p1.

## References

[1] Feldman EL, Nave KA, Jensen TS, Bennett DLH (2017). New horizons in diabetic neuropathy: Mechanisms, bioenergetics, and pain. Neuron.

[2] Jayaraj ND (2018). Reducing CXCR4-mediated nociceptor hyperexcitability reverses painful diabetic neuropathy. J. Clin. Invest..

[3] Sima, A.A., Calvani, M., Mehra, M., Amato, A., & Acetyl-L-Carnitine Study Group. Acetyl-L-carnitine improves pain, nerve regeneration, and vibratory perception in patients with chronic diabetic neuropathy: an analysis of two randomized placebo-controlled trials. *Diabetes Care* **28**, 89-94 (2005). 10.2337/diacare.28.1.89 15616239

[4] Galer BS, Gianas A, Jensen MP (2000). Painful diabetic polyneuropathy: epidemiology, pain description, and quality of life. Diabetes Res. Clin. Pract..

[5] Haroutounian S (2014). Primary afferent input critical for maintaining spontaneous pain in peripheral neuropathy. Pain.

[6] Greene DA, Stevens MJ, Obrosova I, Feldman EL (1999). Glucose-induced oxidative stress and programmed cell death in diabetic neuropathy. Eur. J. Pharmacol..

[7] Cheng HT (2013). Increased axonal regeneration and swellings in intraepidermal nerve fibers characterize painful phenotypes of diabetic neuropathy. J. Pain.

[8] Tesfaye S, Boulton AJ, Dickenson AH (2013). Mechanisms and management of diabetic painful distal symmetrical polyneuropathy. Diabetes Care.

[9] Raputova J (2017). Sensory phenotype and risk factors for painful diabetic neuropathy: A cross-sectional observational study. Pain.

[10] Feldman EL (2019). Diabetic neuropathy. Nat. Rev. Dis. Primers.

[11] Greenbaum D, Richardson PC, Salmon MV, Urich H (1964). Pathological observations in six cases of diabetic neuropathy. Brain.

[12] Schmidt RE (1997). Dystrophic axonal swellings develop as a function of age and diabetes in human dorsal root ganglia. J. Neuropathol. Exp. Neurol..

[13] Haberberger RV, Barry C, Dominguez N, Matusica D (2019). Human Dorsal Root Ganglia. Front. Cell. Neurosci..

[14] Sapio MR, Goswami SC, Gross JR, Mannes AJ, Iadarola MJ (2016). Transcriptomic analyses of genes and tissues in inherited sensory neuropathies. Exp. Neurol..

[15] Ray P (2018). Comparative transcriptome profiling of the human and mouse dorsal root ganglia: an RNA-seq-based resource for pain and sensory neuroscience research. Pain.

[16] Wangzhou A (2020). Pharmacological target-focused transcriptomic analysis of native vs cultured human and mouse dorsal root ganglia. Pain.

[17] Schwaid AG, Krasowka-Zoladek A, Chi A, Cornella-Taracido I (2018). Comparison of the Rat and Human Dorsal Root Ganglion Proteome. Sci. Rep..

[18] Davidson S (2014). Human sensory neurons: Membrane properties and sensitization by inflammatory mediators. Pain.

[19] Seok J (2013). Genomic responses in mouse models poorly mimic human inflammatory diseases. Proc. Natl. Acad. Sci. USA.

[20] Love MI, Huber W, Anders S (2014). Moderated estimation of fold change and dispersion for RNA-seq data with DESeq2. Genome Biol..

[21] Giulietti, A. *et al*. Monocytes from type 2 diabetic patients have a pro-inflammatory profile. 1,25 Dihydroxyvitamin D(3) works as anti-inflammatory. *Diabetes Res. Clin. Pract.* **77**,47–57 (2007). 10.1016/j.diabres.2006.10.007 17112620

[22] Spranger J (2003). Inflammatory cytokines and the risk to develop type 2 diabetes: results of the prospective population-based European Prospective Investigation into Cancer and Nutrition (EPIC)-Potsdam Study. Diabetes.

[23] Bartley EJ, Fillingim RB (2013). Sex differences in pain: a brief review of clinical and experimental findings. Br. J. Anaesth..

[24] Thakur M (2014). Defining the nociceptor transcriptome. Front. Mol. Neurosci..

[25] Ara J, Bannerman P, Shaheen F, Pleasure DE (2005). Schwann cell-autonomous role of neuropilin-2. J. Neurosci. Res..

[26] Harvey VL, Caley A, Müller UC, Harvey RJ, Dickenson AH (2009). A Selective Role for alpha3 Subunit Glycine Receptors in Inflammatory Pain. Front. Mol. Neurosci..

[27] Hill, R.Z. *et al*. The signaling lipid sphingosine 1-phosphate regulates mechanical pain. *Elife* **7** (2018). 10.7554/eLife.33285 PMC5896955 29561262

[28] Tavares-Ferreira, D. *et al*. Spatial transcriptomics reveals unique molecular fingerprints of human nociceptors. *bioRxiv* 02.06.430065 (2021).

[29] Rosas OR (2011). Expression and activation of ephexin is altered after spinal cord injury. Dev. Neurobiol..

[30] Mitchell K (2014). Nociception and inflammatory hyperalgesia evaluated in rodents using infrared laser stimulation after Trpv1 gene knockout or resiniferatoxin lesion. Pain.

[31] Ellis A, Bennett DL (2013). Neuroinflammation and the generation of neuropathic pain. Br. J. Anaesth..

[32] Zhai X (2016). Cell Activation is Associated with Type 2 Diabetes Development in Obese Subjects. Cell Physiol. Biochem..

[33] Wang S (2018). S100A8/A9 in Inflammation. Front. Immunol..

[34] Mitchell K (2008). Localization of S100A8 and S100A9 expressing neutrophils to spinal cord during peripheral tissue inflammation. Pain.

[35] Chernov AV (2015). The calcium-binding proteins S100A8 and S100A9 initiate the early inflammatory program in injured peripheral nerves. J. Biol. Chem..

[36] Chevrier S (2009). CD93 is required for maintenance of antibody secretion and persistence of plasma cells in the bone marrow niche. Proc. Natl. Acad. Sci. USA.

[37] Cancello R (2011). Urokinase plasminogen activator receptor in adipose tissue macrophages of morbidly obese subjects. Obes. Facts.

[38] Kwilasz AJ, Grace PM, Serbedzija P, Maier SF, Watkins LR (2015). The therapeutic potential of interleukin-10 in neuroimmune diseases. Neuropharmacology.

[39] Kitade H (2012). CCR5 Plays a Critical Role in Obesity-Induced Adipose Tissue Inflammation and Insulin Resistance by Regulating Both Macrophage Recruitment and M1/M2 Status. Diabetes.

[40] Daousi C (2004). Chronic painful peripheral neuropathy in an urban community: a controlled comparison of people with and without diabetes. Diabet. Med..

[41] Jensen TS (2006). New perspectives on the management of diabetic peripheral neuropathic pain. Diab. Vasc. Dis. Res..

[42] Calcutt NA (2002). Potential mechanisms of neuropathic pain in diabetes. Int. Rev. Neurobiol..

[43] Martin SL, Reid AJ, Verkhratsky A, Magnaghi V, Faroni A (2019). Gene expression changes in dorsal root ganglia following peripheral nerve injury: roles in inflammation, cell death and nociception. Neural Regen. Res..

[44] Hotamisligil GS (2017). Inflammation, metaflammation and immunometabolic disorders. Nature.

[45] Binshtok AM (2008). Nociceptors are interleukin-1beta sensors. J. Neurosci..

[46] Black BJ (2018). Adult Mouse Sensory Neurons on Microelectrode Arrays Exhibit Increased Spontaneous and Stimulus-Evoked Activity in the Presence of interleukin-6. J. Neurophysiol..

[47] Hur J (2011). The identification of gene expression profiles associated with progression of human diabetic neuropathy. Brain.

[48] Yang D, Han Z, Oppenheim JJ (2017). Alarmins and immunity. Immunol. Rev..

[49] Hinder LM (2018). Transcriptional networks of progressive diabetic peripheral neuropathy in the db/db mouse model of type 2 diabetes: An inflammatory story. Exp. Neurol..

[50] Kiguchi N, Kobayashi D, Saika F, Matsuzaki S, Kishioka S (2017). Pharmacological Regulation of Neuropathic Pain Driven by Inflammatory Macrophages. Int. J. Mol. Sci..

[51] Kiguchi N (2012). Epigenetic augmentation of the macrophage inflammatory protein 2/C-X-C chemokine receptor type 2 axis through histone H3 acetylation in injured peripheral nerves elicits neuropathic pain. J. Pharmacol. Exp. Ther..

[52] Yu X (2020). Dorsal root ganglion macrophages contribute to both the initiation and persistence of neuropathic pain. Nat. Commun..

[53] Renthal W (2020). Transcriptional Reprogramming of Distinct Peripheral Sensory Neuron Subtypes after Axonal Injury. Neuron.

[54] Waxman SG, Zamponi GW (2014). Regulating excitability of peripheral afferents: emerging ion channel targets. Nat. Neurosci..

[55] Papanas N, Ziegler D (2012). Prediabetic neuropathy: does it exist?. Curr. Diab. Rep..

[56] Tesfaye S (2016). Diabetic peripheral neuropathy may not be as its name suggests: evidence from magnetic resonance imaging. Pain.

[57] Kapur D (2003). Neuropathic pain and diabetes. Diabetes Metab. Res. Rev..

[58] Lerner AG (2012). IRE1α induces thioredoxin-interacting protein to activate the NLRP3 inflammasome and promote programmed cell death under irremediable ER stress. Cell Metab..

[59] Inceoglu B (2015). Endoplasmic reticulum stress in the peripheral nervous system is a significant driver of neuropathic pain. Proc. Natl. Acad. Sci. USA.

[60] Barragán-Iglesias P (2019). Activation of the integrated stress response in nociceptors drives methylglyoxal-induced pain. Pain.

[61] Kamiya H, Zhang W, Sima AA (2006). Degeneration of the Golgi and neuronal loss in dorsal root ganglia in diabetic BioBreeding/Worcester rats. Diabetologia.

[62] Bennett DA (2018). Religious Orders Study and Rush Memory and Aging Project. J Alzheimer’s Dis..

[63] The Gene Ontology Consortium (2019). The Gene Ontology Resource: 20 years and still GOing strong. Nucleic Acids Res..

[64] Crowe, A.R., & Yu, W. Semi-quantitative determination of protein expression using immunohistochemistry staining and analysis: an integrated protocol. *Bio. Protoc.* **9**, e3465 (2019). 10.21769/BioProtoc.3465 PMC6924920 31867411

[65] Wang F (2021). RNAscope: a novel in situ RNA analysis platform for formalin-fixed, paraffin-embedded tissues. J. Mol. Diagn..

